# Effect of dietary fat source on the composition of the cecal microbiome in maturing broiler chicken

**DOI:** 10.3389/fmicb.2024.1462757

**Published:** 2024-11-27

**Authors:** Vidya V. Jadhav, Yewande Fasina, Paul C. Omaliko, Jian Han, Scott H. Harrison

**Affiliations:** ^1^Department of Biology, North Carolina Agricultural and Technical State University, Greensboro, NC, United States; ^2^Department of Animal Sciences, North Carolina Agricultural and Technical State University, Greensboro, NC, United States

**Keywords:** broiler chicken, chicken microbiome, 16S rRNA gene, microbial composition, microbial function, dietary fat, polyunsaturated fat, saturated fat

## Abstract

Diet has been found to significantly influence gut microbiota throughout various life stages, and gut microbiota have been increasingly shown to influence host physiology, health, and behavior. This study uses 16S rRNA sequencing to examine the effects of six different fat-supplemented diets (canola oil, coconut oil, fish oil, flaxseed oil, lard, and olive oil) on broiler chicken cecal microbial composition and predicted function in comparison with a common and inexpensive fat source (poultry fat). Groups of broilers were fed each of these diets and then evaluated on day 41 and day 55 of age. For both 41- and 55-day samples, *Firmicutes* and *Bacteroidetes* phyla were the dominant bacteria in the ceca accounting for 99% of the microbial community. Across the 41- and 55-day samples, treatment time was associated with a stronger and more significant microbiota shift (*p* < 0.001) than differences in dietary treatment alone (*p* = 0.117), but dietary treatment combined with treatment time is a significant factor as well (*p* = 0.047). Sparse partial least squares discriminant analysis was used to explore the more discriminating taxa for each treatment group. For identified species, butyrate production appears to be affected in a diet-specific manner, with many butyrate-producing species being evident for the fish-based diet at day 41 and a few of these species for the flaxseed-based diet at day 55. Predicted functions, as conducted with PICRUSt2, were significant for comparisons between the control and the flaxseed-based dietary treatment group at day 55, with indications of host health benefit for the flaxseed-based diet. Predicted functions found to be significant were for enzymes and pathways such as propionate CoA ligase, aminobutyraldehyde dehydrogenase, vitamin B12-transporting ATPase, thiamine kinase, acetylneuraminate epimerase, and L-tryptophan biosynthesis. This study provides insight surrounding specific dietary fat-based treatments to be investigated further and highlights the importance of polyunsaturated fat sources in poultry feed that may offer a favorable cecal microbial modulation compared to saturated fat sources.

## 1 Introduction

Broiler chickens are crucial to the agribusiness sector, driven by the rising demand for poultry meat, which is projected to reach 103.3 million tons globally in 2024 (Precedence Research, 2023). Consequently, the health and performance of broiler chickens hold substantial economic importance. However, the ban on antibiotics in poultry production has intensified the challenge of disease control, underscoring the need for alternative strategies to support gut health and enhance the birds’ natural defenses against pathogenic bacteria (Zhu et al., 2021). One of the most critical factors in achieving optimal health and productivity is diet, which plays a pivotal role in shaping the gut microbial community. Consequently, the composition of the gut microbiota is essential for maintaining gut integrity, increasing pathogen resistance, modulating immune response, and optimizing nutrient absorption, all of which contribute to overall bird health and performance (Nova et al., 2022; Rychlik, 2020).

Dietary fats are included in the poultry feed to support rapid growth and meet the energy demands of fast-growing commercial broilers which typically reach market weight within a short life span. Apart from a concentrated energy source, dietary fats also influence microbial activity in the gut. Dietary fats are primarily metabolized by the host system, while the undigested fats that reach the hindgut are further metabolized by gut microbes to produce energy (Barszcz et al., 2024). This microbial fermentation contributes to fat metabolism. In addition, the specific fatty acid profile of dietary fats influences bile secretion and fat digestibility, ultimately shaping the gut microbial composition and gut health (Baião and Lara, 2005; Józefiak et al., 2014; Schoeler and Caesar, 2019; Zeitz et al., 2015). Therefore, it is essential to understand the effect of different fat sources rich in polyunsaturated fatty acids (PUFAs), monounsaturated fatty acids (MUFAs), and saturated fatty acids (SFAs) on gut microbial composition.

Dietary fats rich in PUFAs, particularly alpha-linolenic acid (omega-3 C18:3) and docosahexaenoic acid (omega-3 C22:6), have been found to encourage gut microbial diversity and enrich beneficial gut microbes in humans and some animals (Watson et al., 2017). However, this general effect has shown variability and inconsistency across multiple studies (Wolters et al., 2019; Abulizi et al., 2019). Beyond an increase in gut microbial diversity, PUFA supplementation in mouse models has been linked with increased levels of short-chain fatty acids (SCFAs), and varied effects on immune response and gut health (Fu et al., 2021; Noriega et al., 2016; Salsinha et al., 2024; Yang et al., 2017; Abulizi et al., 2019). In addition, in chickens, a diet rich in PUFAs has been reported to enrich *Lactobacilli, Tenericutes*, and *Bacteroides* species (Alzueta et al., 2003; Neijat et al., 2020). Conversely, the dietary inclusion of SFA sources such as lard and tallow has been associated with adverse health outcomes. Lard diets in chickens have led to increased pathogenic *C. perfringens* in the small intestine. Moreover, the effects of microbiome composition have been found to vary with and be dependent on the duration of exposure to dietary treatments and the age of the chicken (Knarreborg et al., 2002).

Numerous studies have explored the effects of dietary fat sources on the growth performance of broiler chickens; however, further research is warranted to examine this impact in conjunction with the gut microbiome (Al-Hilali, 2018; Aziza et al., 2014; Kamran et al., 2020). Using genomic data to illuminate the composition and potential functional role of the microbiome in response to dietary fat can optimize poultry feed and enhance our understanding of the specific influence of gut microbes on host physiology, health, and behavior. Our research employs amplicon sequencing to examine the effects of six different dietary fat sources on broiler cecal microbial composition and predicted function, comparing them to the most common and inexpensive fat source, poultry fat. The six dietary fat sources include PUFA-rich fat sources flaxseed oil and fish oil, MUFA-rich fat sources canola oil and olive oil, and SFA-rich fat sources lard and coconut oil.

Our study investigates the microbial composition of cecal content as the cecum has high microbial diversity and activity compared to other sections of the GI tract. We further evaluate these effects in broiler chickens at 41 and 55 days of age, a critical period surrounding the typical time of harvesting. This approach aims to provide a comprehensive understanding of how different dietary fats influence the gut microbiome and, consequently, broiler health and productivity.

## 2 Materials and methods

### 2.1 Animals and dietary treatment

Animal care and use procedures were approved by the Institutional Animal Care and Use Committee of North Carolina Agricultural and Technical State University (IACUC #20-004.0) prior to the commencement of the study. A total of 560 one-day-old male broiler chicks (Ross 708) were obtained from a commercial chicken hatchery and housed at the Poultry Research Unit of the North Carolina Agricultural and Technical State University (Greensboro, NC, USA). Chicks were randomly assigned in equal numbers into seven experimental treatment groups. The experimental treatments consisted of the conventional corn-soybean meal (SBM) control diet containing 3% poultry fat (CN) and a corn-SBM basal diet in which lard (LA), coconut oil (CC), olive oil (OL), canola oil (CA), fish oil (FI), or flaxseed oil (FL) were alternatively incorporated at a 3% level. The experimental diets were formulated to be isocaloric and were manufactured at the North Carolina State University Feed Education Unit (Raleigh, NC, USA). The six fat types were purchased commercially from Jedwards International, Inc. (Braintree, MA, USA). The fatty acid composition of each fat type was determined at the Eurofins Nutrition Analysis Center (Des Moines, IA) before incorporating them into the experimental diets (Supplementary Table 1).

Each treatment consisted of 5 replicate pens, with 16 chickens randomly assigned to each pen. The experiment was carried out for 56 days. From day 1 to day 21, the chicks were housed in battery cages (Alternative Design Manufacturing and amp Supply Inc., Siloam Springs, AR). Each battery cage had a nipple drinker to supply water and a feeder tray which was adjusted in height for reach according to the progressive growth of the chicks. At 21 d, the chicks were transferred to representative fresh pine savings liter floor pens that were equipped with a hanging feeder and a bell drinker. Chickens were fed ad *libitum* and allowed free access to water throughout the experiment. The bird housing was maintained at 92°F from day 1 to day 7 and then 87°F from day 8 to day 21. Subsequently, it was reduced to 77°F to day 55. The photoperiod consisted of 23L:1D from d1 to 7, 20L:4D from d8 to 21 at 30 lux, and then 24L:0D from day 22 to day 56.

Chicks were given starter diets (as crumbled pellets) from one-day-old to 3 weeks (i.e., days 1-20) followed by a grower diet (pellets) until 6 weeks (i.e., days 21-41) and then a finisher diet fed as pellets from 6 weeks to 8 weeks (i.e., days 42-56). The diets were formulated to meet or slightly exceed nutritional requirements based on the recommendations in the Ross Broiler Nutrition Specification Handbook (Aviagen, 2022) (Supplementary Tables 2–4).

At days 41 and 55 of age, chickens were randomly taken from each treatment group (one bird per replicate) and were humanely euthanized using CO_2_ asphyxiation. Cecal content was collected using sterile instruments in a clean environment immediately after dissection. The samples were placed into sterile screwcap tubes, promptly snap-frozen in liquid nitrogen, and subsequently stored at −80°C until further processing. Cecal content from five birds per treatment group was used for the subsequent DNA extraction for 16S rRNA gene sequencing.

### 2.2 DNA extraction and 16S rRNA gene sequencing

For genomic DNA extraction, 200 mg of cecal contents was utilized. The frozen cecal content was slowly thawed on ice, and DNA extraction was carried out using the QIAamp PowerFecal Pro DNA Kit (Qiagen Inc., United States), following the manufacturer’s protocol (Emami et al., 2020; Lu et al., 2020; Williams and Athrey, 2020). This protocol includes mechanical (bead beating) and enzymatic steps for maximum recovery of microbial genomic DNA. The extracted DNA was assessed for quality (A260/A280 and A260/A230) and quantity using the DeNovix DS-11 Series instrument and QuantiFluor^®^ dsDNA System (Promega), respectively. The extracted DNA was stored at −20°C until further use.

The V4 hypervariable region of the 16S rRNA gene was amplified from extracted DNA samples using the forward primer 515F (5′-GTGCCAGCMGCCGCGGTAA-3′) and reverse primer 806R (5′-GGACTACHVGGGTWTCTAAT-3′) (Emami et al., 2020; Plata et al., 2022; Williams and Athrey, 2020). Subsequent sequencing of the amplicons was carried out at Argonne National Laboratory using Illumina MiSeq with a 250×250 cycle configuration for 16S rRNA.

### 2.3 Cecal microbiota compositional analysis and statistical analysis

The raw sequencing data obtained from the sequencing center were processed and analyzed using the QIIME2 pipeline (version 2023.5.1) (Estaki et al., 2020). The DADA2 pipeline was used further for quality filtering and denoising the output (parameters: p-trunc-len-f 233 and p-trunc-len-r 226) and selection of sequences with a quality score > 30, which were grouped into Amplicon Sequence Variants (ASVs) (Callahan et al., 2016). Taxonomic assignment of processed reads/sequences was carried out using a QIIME2 pre-trained naive Bayesian classifier trained on Greengenes2 (updated on 2022.10) reference database (Bokulich et al., 2018).

Following the taxonomic assignment, data analysis was conducted with R (version 4.3.0) and the Bioconductor R package phyloseq (version 1.44.0). The QIIME2 output was exported to the phyloseq object using the R package qiime2R (version 0.99.6). A total of 3,237 ASVs were identified, comprising 26 phyla, 33 classes, 68 orders, 110 families, 296 genera, and 248 species. Furthermore, all ASVs with a count of more than two and present in more than 5% of samples were retained for analysis. Pathway abundance prediction was performed using PICRUSt2.

The ASV absolute count data were transformed into relative abundance specifically for taxonomic bar plots. ASV absolute count data were otherwise transformed via central log ratio. Alpha diversity indices were estimated for all treatment groups at day 41 and day 55 and were measured using the plot_richness function from the phyloseq R package. The principal coordinate analysis (PCoA) was performed using Euclidean and Bray–Curtis distance measures with the ecodist R package. Beta diversity was estimated using the Aitchison (Euclidean on a center log-ratio transformation count data) and Bray–Curtis distance to analyze compositional differences among the treatment groups using non-parametric multivariate ANOVA based on dissimilarities (adonis2) with 1,000 random permutations package used vegan (version 2.6–4).

To identify the most discriminating ASVs among the treatment groups compared to others, the mixOmics R package (version 6.24.0) was utilized. From this package, sparse partial least squares discriminant analysis (sPLS-DA) was used for discriminating taxa identification (Cao et al., 2011). sPLS-DA is a supervised classification method that selects the most discriminating ASVs from the group using the LASSO penalization method. The method uses an iterative cross-validation method to select the most stable ASVs. Partial least square-discriminant analysis (PLS-DA) is first used to identify a number of components to be then used for sPLS-DA analysis; final sparse PLS-DA models were built based on 6 and 7 components for day 41 and day 55 samples, respectively. The model performance was analyzed using 5-fold cross-validation using Mahalanobis distance with 1,000 iterations. Differentially abundant taxa selected by sPLS-DA analysis were then tested for significant differences across treatment groups using the non-parametric Kruskal–Wallis test followed by Dunn’s test for multiple comparisons. ALDEx2 was employed to detect differentially abundant pathways from the PICRUSt2-predicted pathways between treatment groups. Multiple comparisons between treatment groups were tested using Dunn’s test.

## 3 Results

### 3.1 Fatty acid composition of dietary treatment groups

The fatty acid composition for the experimental treatment diets is shown in Supplementary Table 1. The PUFA levels were highest in the flaxseed oil group (64.33%), followed by fish oil (37.52%) and canola oil group (24.17%), while low levels of PUFAs were observed in the coconut oil (0.78%), olive oil (6.59%), and lard group (14.23%). In terms of SFA, higher levels were observed in the coconut oil (85.58%) and the lard group (42.89%). The olive oil group has the highest levels of MUFAs (71.93%), followed by the canola oil group (61.26%).

### 3.2 Cecal microbiota compositional analysis

**Figure 1:**
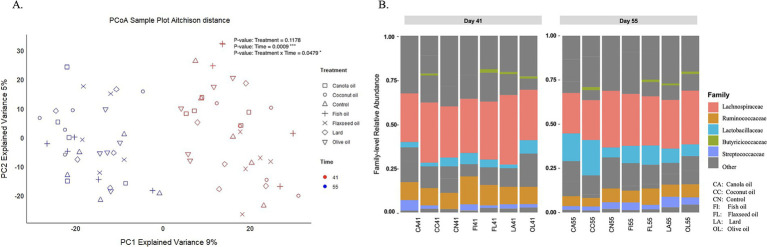
Cecal microbial composition. **(A)** Principal coordinate analysis **(**PCoA) based on Aitchison distances with points in the plot representing the cecal microbiota of individually sampled chickens, with sample categories shown by point shape (dietary fat type) and colors (age in days). The *p*-values shown are based on PERMANOVA across factors of treatment, time, and treatment–time interaction (*p < 0.05; ***p < 0.001). (**B)** Stacked bar plot showing mean relative abundance of top abundant families in day 41 and day 55 cecal samples across treatment groups. Highly abundant biologically important families are shown with colors, while other less important families are represented in gray.

Figure 1A shows the principal coordinate analysis of day 41 and day 55 samples, illustrating distinct clusters based on age, indicating differences in their microbial composition over time. The relative abundance of microbial communities between 41- and 55-day samples revealed that *Firmicutes* and *Bacteroidetes* phyla were the dominant bacteria in the ceca, comprising 99% of the microbial community, out of 26 total detected phyla (Supplementary Figure 1). Figure 1B illustrates relative abundance plots highlighting the top important families from both day 41 and day 55 samples.

### 3.3 Microbial diversity analysis

Alpha diversity was calculated using the Chao1, Shannon, ACE, and Simpson indices to evaluate species richness and evenness among the treatments for samples at days 41 and 55 (Supplementary Figure 2). Statistical analysis of the genus-level Shannon index between 41- and 55-day samples across the treatment groups and the control group showed no significant difference in diversity. The fish group showed a slight trend in microbial diversity at day 55 compared to day 41 (*p* = 0.094) (Supplementary Table 5). Microbial compositional differences between the treatment groups were analyzed using permutational analysis of variance (PERMANOVA) across factors of treatment, time, and treatment–time interaction (Table 1). There is a very significant effect of age (day 41 and day 55) on microbial composition across the treatment groups (*p* < 0.001). The interaction of treatment and time was also found to be significant (*p* = 0.047).

**Table 1:** Permuted multivariate analysis of variance (PERMANOVA) testing for differences in community structure.

Factors	Degree of freedom	Aitchison distance	
		Pseudo-F	*P*-value^a^
Treatment	6 (CA, CC, CN, FI, FL, LA, OL)	1.0761	0.1178
Time	1 (day 41, day 55)	6.2195	0.0009 ***
Treatment * Time	6 (CA, CC, CN, FI, FL, LA, OL)	1.4691	0.0479 *

CA, canola oil; CC, coconut oil; CN, control; FI, fish oil; FL, flaxseed oil; LA, lard; OL, olive oil. ^a^ *p < 0.05; **p < 0.01; ***p < 0.001.

To further investigate the effect of treatment compared to the control group (poultry fat), a pairwise comparison between the treatment and control group was conducted using pairwise Adonis. However, the microbial composition of the day 41 control group did not significantly differ from other treatment groups based on both adjusted and unadjusted *p*-values (Table 2). Among non-control group comparisons, significance was observed in unadjusted *p*-values, particularly involving the flaxseed oil diet. At day 55, the control group exhibited compositional differences compared to the coconut, flaxseed, and olive oil-supplemented groups based on unadjusted *p*-values (Table 3).

**Table 2:** ASV-level pairwise comparison for day 41.

	Pairs	Df	Sums of Sqs	*F* model	*R* ^2^	*P*-value^a^	*P* adjusted (Bonferroni)
1	CN vs. CA	1	2562.66	0.85	0.10	0.84	1.00
2	CN vs. CC	1	3358.87	1.07	0.12	0.25	1.00
3	CN vs. FI	1	3765.06	1.30	0.14	0.10	1.00
4	CN vs. FL	1	3717.62	1.10	0.12	0.22	1.00
5	CN vs. LA	1	3011.73	0.94	0.10	0.71	1.00
6	CN vs. OL	1	2842.38	0.88	0.10	0.89	1.00
7	FL vs. CA	1	4659.93	1.45	0.15	0.01**	0.23
8	FL vs. FI	1	5226.00	1.69	0.17	0.04*	0.80
9	FL vs. OL	1	4278.92	1.25	0.13	0.03*	0.59

CA, canola oil; CC, coconut oil; CN, control; FI, fish oil; FL, flaxseed oil; LA, lard; OL, olive oil. ^a^ *p < 0.05; **p < 0.01; ***p < 0.001.

**Table 3:** ASV-level pairwise comparison for day 55.

	Pairs	Df	Sums of Sqs	*F* model	*R* ^2^	*P*-value^a^	*P* adjusted (Bonferroni)
1	CN vs. CA	1	2981.35	1.02	0.11	0.40	1.00
2	CN vs. CC	1	3919.74	1.41	0.15	0.01**	0.25
3	CN vs. FI	1	3214.92	1.14	0.12	0.06	1.00
4	CN vs. FL	1	3477.75	1.21	0.13	0.05*	1.00
5	CN vs. LA	1	3073.39	1.05	0.12	0.35	1.00
6	CN vs. OL	1	3474.35	1.20	0.13	0.04*	0.90
7	CC vs. OL	1	3138.59	1.16	0.13	0.09	1.00

CA, canola oil; CC, coconut oil; CN, control; FI, fish oil; FL, flaxseed oil; LA, lard; OL, olive oil. ^a^ *P < 0.05; **P < 0.01; ***P < 0.001.

### 3.4 Sparse partial least squares discriminant analysis (sPLS-DA)

**Figure 2:**
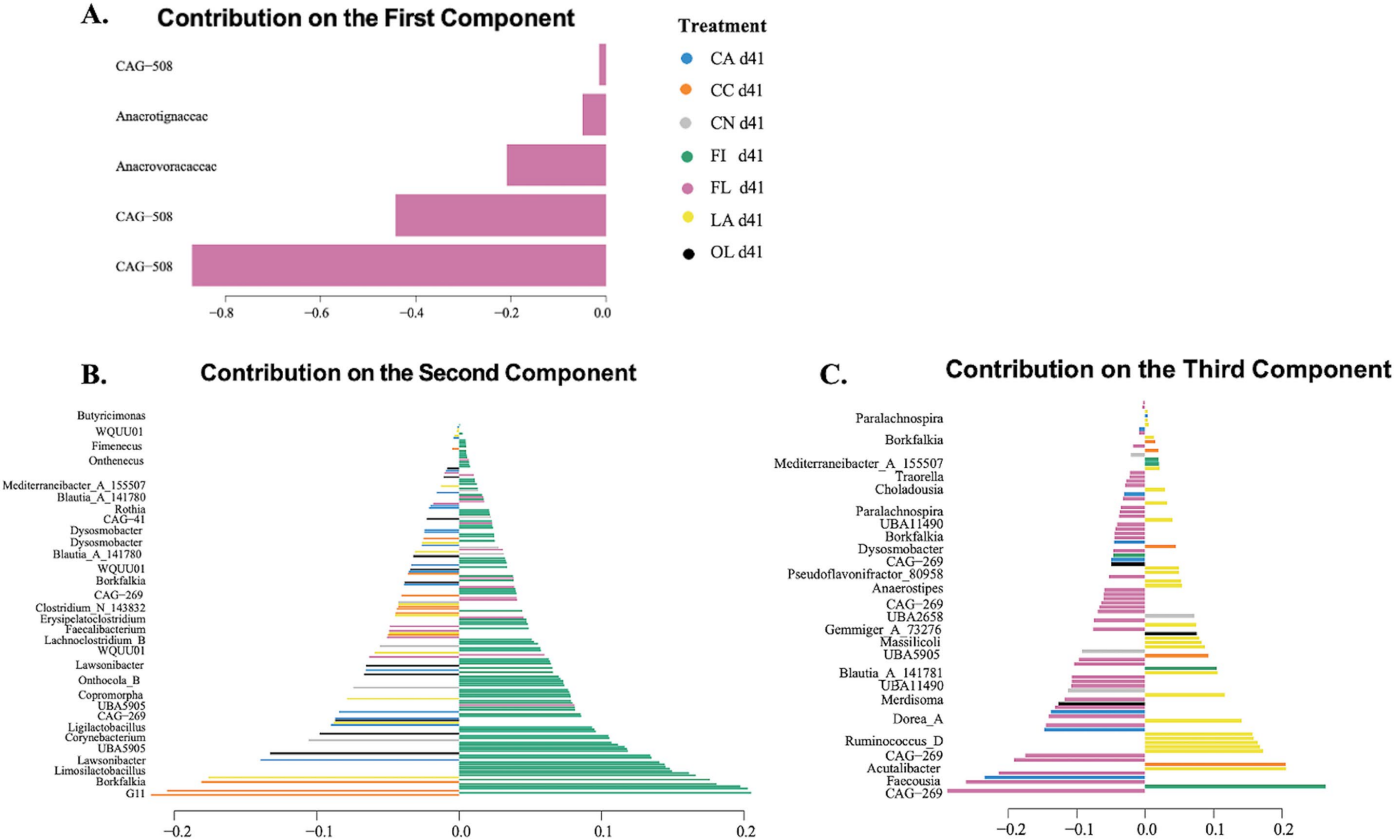
Sparse partial least squares discriminant analysis (sPLS-DA) loading plots for day 41 samples. The length of the bar represents the loading weight, and the sign of the loading weights indicates the direction of the contribution to the component. The color code indicates the dietary fat type. Loading plots and taxonomic levels shown are for: **(A)** component 1 with family-level taxonomic information; **(B)** component 2 with genus-level taxonomic information; and **(C)** component 3 with genus-level taxonomic information. CA, canola oil; CC, coconut oil; CN, control; FI, fish oil; FL, flaxseed oil; LA, lard; OL, olive oil.

The sPLS-DA model-based approach was used to evaluate the most discriminating taxa across the treatment groups for 41- and 55-day samples. Figure 2 displays sPLS-DA loading plots where the length of the bar represents the loading weight; longer bars represent greater importance, while the color of the bars indicates the treatment group (see Supplementary material for a full listing of taxa and sPLS-DA values). The loading plot displays the most differentiating features or taxa on the selected components. For component 1, the most important taxa (family level) were primarily specific to the flaxseed (FL: pink) group. For component 2, the most important taxa (genus level) were primarily specific to the fish oil (FI: green) group. For component 3, the most important taxa (genus level) were primarily specific to the flaxseed and lard (FL: pink; LA: yellow) groups.

**Figure 3:**
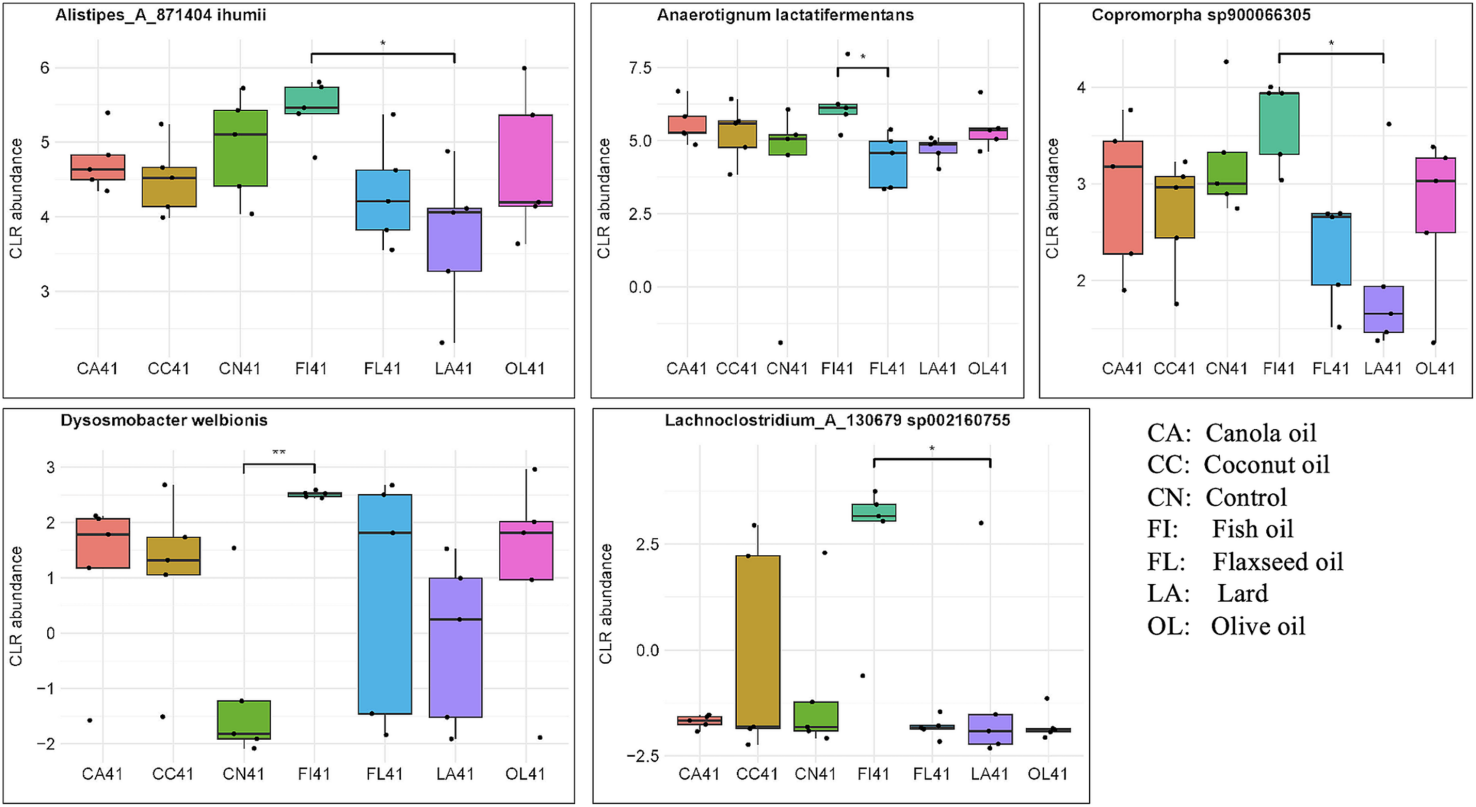
Abundance of biologically important species having significant differences across the dietary fat groups for day 41 samples. Significance was evaluated with the Kruskal–Wallis test followed by Dunn’s test for multiple comparisons (*p < 0.05; **p < 0.01; ***p < 0.001). Taxa evaluated were identified by sPLS-DA modeling.

There were 424 taxa selected by six components of the sPLS-DA model for 41-day samples. Out of these taxa, 46 were found to be significant for differential abundance using the non-parametric Kruskal–Wallis test. Figure 3 and Supplementary Table 6 display several taxa known to be important for host gut health that were found to differ across the dietary fat treatment groups. Notably, *Dysosmobacter welbionis* was enriched in the fish oil group, compared to the control (*p* = 0.009), *Alistipes_A_871404 ihumii* was higher in the fish oil group than in the lard group (*p* = 0.014), *Anaerotignum lactatifermentans* showed differences when compared to flaxseed oil (*p* = 0.047), and *Copromorpha sp900066305* showed differences when compared to the lard group (*p* = 0.014). Kruskal-Wallis test evaluation for *p* < 0.10 would also include *Sellimonas intestinalis* (*p* = 0.047) and *Lachnoclostridium_A_130679 sp002160755* (*p* = 0.040) species compared to the lard group as being enriched in the fish oil group.

**Figure 4:**
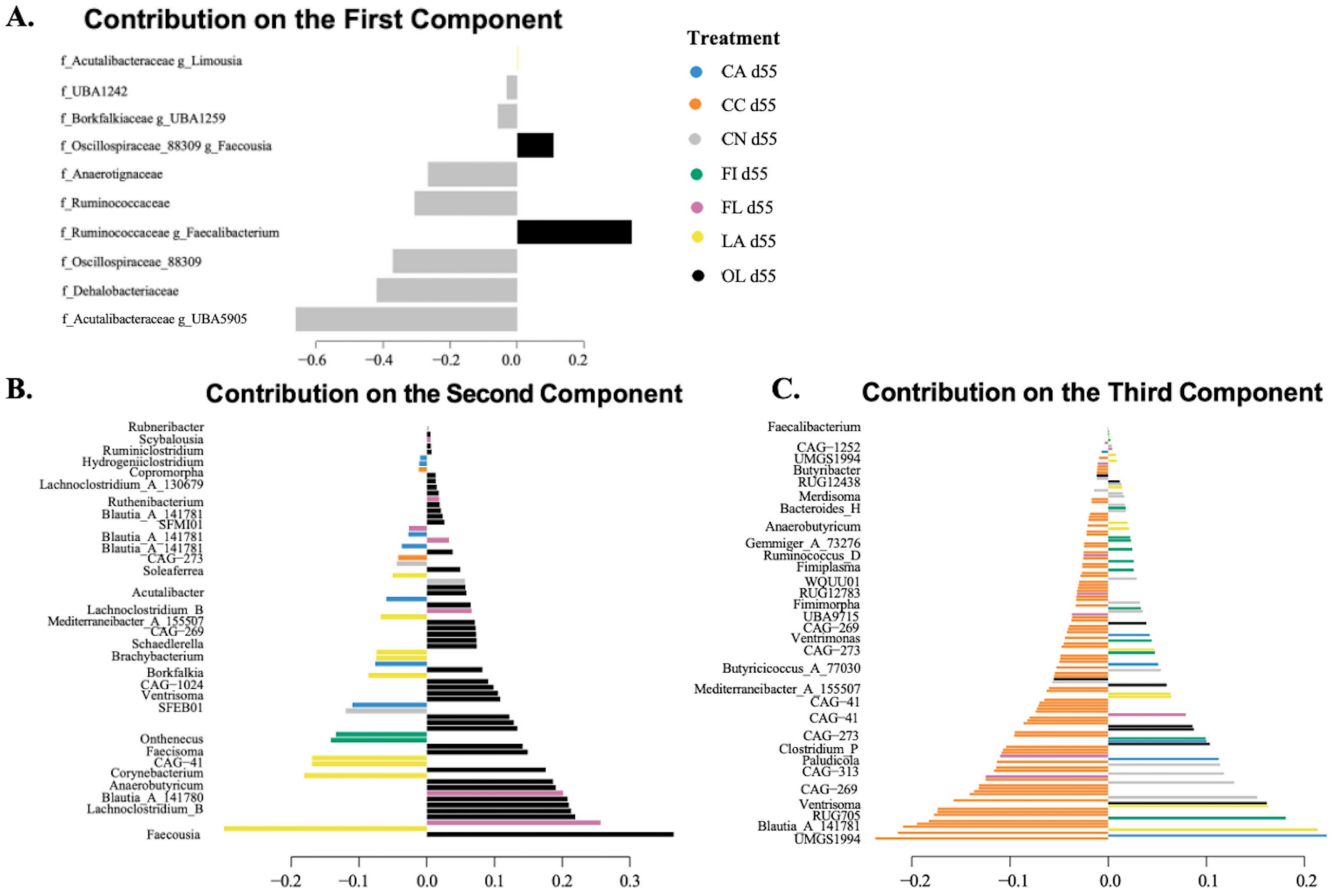
Sparse partial least squares discriminant analysis (sPLS-DA) loading plots for day 55 samples. The length of the bar represents the loading weight, and the sign of the loading weights indicates the direction of the contribution to the component. Loading plots and taxonomic levels shown are for: **(A)** component 1 with family and genus-level taxonomic information; **(B)** component 2 with genus-level taxonomic information; and **(C)** component 3 with genus-level taxonomic information. CA, canola oil; CC, coconut oil; CN, control; FI, fish oil; FL, flaxseed oil; LA, lard; OL, olive oil.

The sPLS-DA loading plots for day 55 samples (Figure 4) indicate that, for component 1, the most important taxonomic genera were primarily specific to the control (CN: gray) group. For component 2, the most important taxa were primarily specific to the olive oil (OL: black) group. For component 3, the most important taxa were primarily specific to the coconut oil (CC: orange) group.

**Figure 5:**
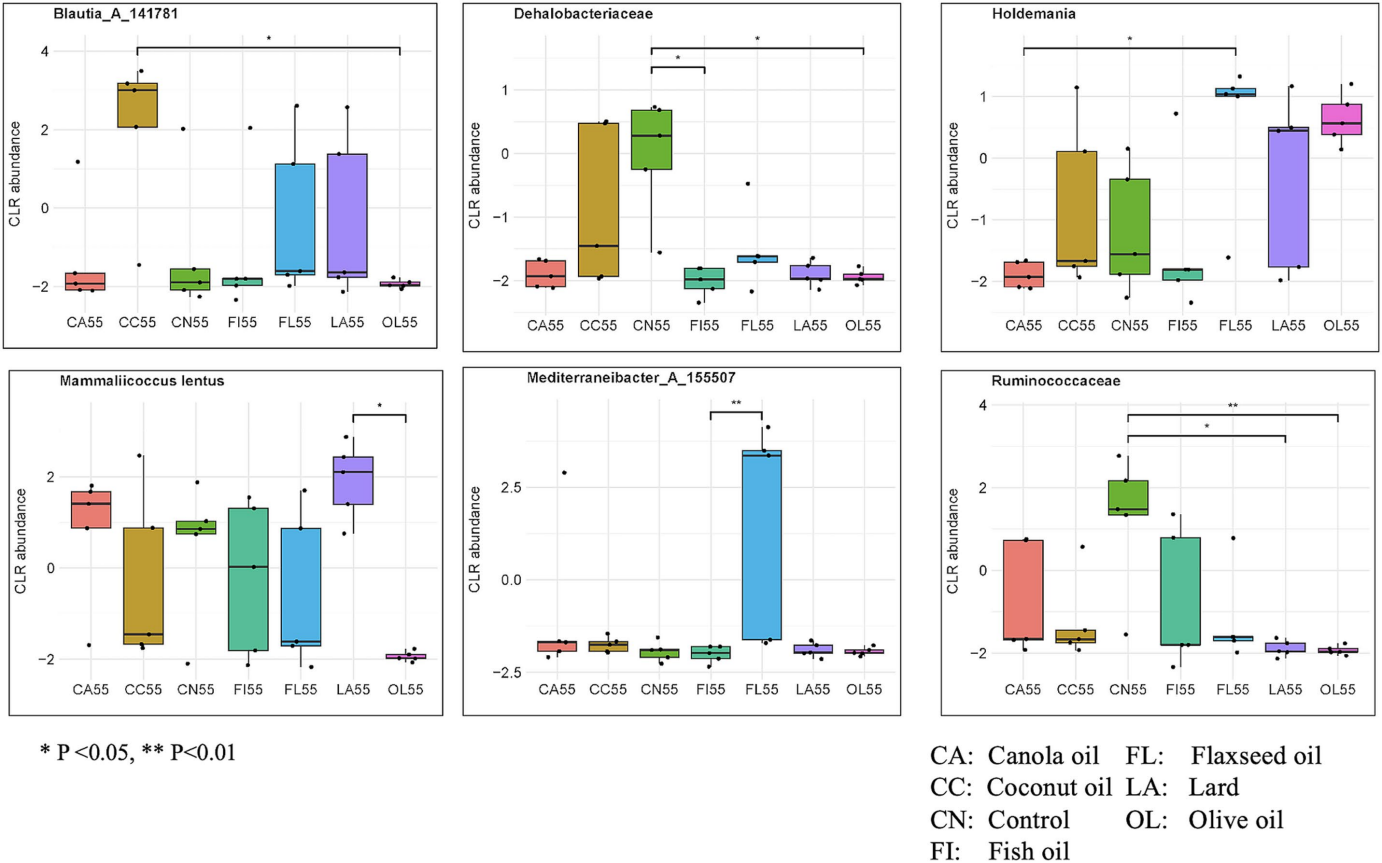
Abundance of biologically important species having significant differences across the dietary fat groups for day 55 samples. Significance was evaluated using the Kruskal–Wallis test followed by Dunn’s test for multiple comparisons (*p < 0.05; **p < 0.01; ***p < 0.001). Taxa evaluated were identified using sPLS-DA modeling.

For the 55-day samples, 606 taxa were selected by 7 components of the sPLS-DA model. There were 53 taxa found to be significant for differential abundance using the non-parametric Kruskal–Wallis test. Figure 5 and Supplementary Table 6 display several taxa important for host gut health. Notably, the bacterial genera *Mediterraneibacter_A_155507* showed significantly higher abundance in the flaxseed group than fish oil (*p* = 0.009), while *Holdemania* was more abundant in the flaxseed group than canola oil (*p* = 0.029). Subspecies of *Blautia_A_141781* were found to be most abundant in the coconut oil group compared to the olive oil group (*p* = 0.026), while *Mammaliicoccus lentus* showed greater abundance in the lard group than the olive oil group (*p* = 0.005). The *Ruminococcaceae* family was significantly more abundant in the control group than in the lard group (*p* = 0.012) and the olive oil group (*p* = 0.003). Similarly, the *Dehalobacteriaceae* family was found to be significantly abundant in the control group compared to fish oil (*p* = 0.010) and olive oil (*p* = 0.032).

### 3.5 Functional prediction of cecal microbiota

To investigate the potential function of the cecal microbiome in all treatment groups, we utilized the PICRUSt2 tool. A total of 346 and 352 MetaCyc metabolic pathways were predicted for 41- and 55-day samples, respectively. After analyzing pathway abundances between treatment groups for each day using ALDEx2, 15 and 32 differentially abundant pathways were identified to have significant unadjusted *p*-values (*p* < 0.05) for the 41- and 55-day samples, respectively (Supplementary Figures 3A,B). These pathways were further analyzed for multiple comparisons with adjusted *p*-values. For day 41, L-lysine fermentation to acetate and butanoate pathway was abundant in flaxseed compared to the fish oil group. For day 55, there were multiple pathways differentially abundant specific to the control versus flaxseed comparison. These pathways were enterobactin biosynthesis (*p* = 0.005), superpathway of L-tryptophan biosynthesis (*p* = 0.009), superpathway of heme biosynthesis from glycine (*p* = 0.009), ppGpp biosynthesis (*p* = 0.021), polymyxin resistance (*p* = 0.012), superpathway of lipopolysaccharide biosynthesis (*p* = 0.009), enterobacterial common antigen biosynthesis (p = 0.012), superpathway of (Kdo)2-lipid A biosynthesis (*p* = 0.009), L-arginine degradation II (AST pathway) (*p* = 0.008), superpathway of methylglyoxal degradation (*p* = 0.026), 2-methylcitrate cycle I and II (*p* = 0.005, *p* = 0.007), and sulfoglycolysis (*p* = 0.010) which were found to have higher abundance in the flaxseed oil group than in the control group (Supplementary Figure 3B).

In the case of predicted enzymes, 1782 and 1822 EC enzymes were identified for 41- and 55-day samples, respectively. After analyzing enzyme abundances between treatment groups for each day using ALDEx2, 30 and 168 differentially abundant enzymes were identified to have significant unadjusted *p*-values (*p* < 0.05) for 41- and 55-day samples, respectively. Significantly abundant enzymes identified in the day 41 samples include propanal dehydrogenase (CoA-propanoylating) (*p* = 0.011), which was abundant in the flaxseed oil group, taurine-2-oxoglutarate transaminase (*p* = 0.023) abundant in the olive oil group, and valine–pyruvate transaminase (*p* = 0.019) abundant in the fish oil group. For day 55 samples, 115 enzymes showed significant (*p* < 0.05) differences in gene abundance for the flaxseed oil group compared to the control group. Some of these important enzymes include aminobutyraldehyde dehydrogenase, propionate kinase, formate dehydrogenase-N, L-lactate dehydrogenase (cytochrome), D-lactate dehydratase, riboflavin reductase [NAD(P)H], vitamin B12-transporting ATPase, thiamine kinase, taurine dioxygenase, S-formyl-glutathione hydrolase glutathione transferase, and acetylneuraminate epimerase.

## 4 Discussion

### 4.1 Longer treatment time, diet, and age of the bird affect cecal microbial composition

The PERMANOVA conducted in the study indicates that the age of the bird and the interaction between the age and treatment groups relate to significant microbial compositional differences. This interaction between the age and treatment groups suggests varied effects of treatment on the microbial community composition across these time points. This interaction effect between age and treatment is further supported by the alpha and beta diversity analyses. The genus-level microbial diversity was observed to be increasing with the age of the fish oil group (Supplementary Table 5). In addition, the pairwise comparison for Aitchison distances between treatment and the control groups for different ages has shown distinct microbial composition for treatment and control pairs for day 41 and day 55 (Tables 2, 3). The varying effects of treatment observed at day 41 and day 55, along with the reported interactions with diet, indicate that the different energy and nutrient requirements of broilers at these ages significantly influence their gut microbiomes. This also highlights the differential impacts associated with specific diet treatments.

Earlier studies have demonstrated that the age of broilers significantly influences the cecal microbial composition. The diversity and complexity of the cecal microbiota increase rapidly in the early stages of life, with noticeable changes up to approximately 35–40 days of age (Yin et al., 2023; Takeshita et al., 2021). After this period, the microbial community stabilizes, exhibiting a relatively consistent structure that supports gut health and function (Takeshita et al., 2021; Li et al., 2018).

While dietary interventions in broiler chickens can influence microbiome composition, their effects are generally less pronounced compared to the substantial microbial shifts driven by age (Knarreborg et al., 2002). In our study, the limited sample size per treatment group across the multiple dietary interventions evaluated may have impeded discernment of effects. However, even with larger sample sizes spanning fewer diets, significant differences may not be always found. For instance, a study investigating the effects of lauric acid over 35 days in 180 broiler chickens found no significant shifts in microbiota (Zeitz et al., 2015). A study investigating olive extract supplementation in 306 chickens found no effect in measures of cecal microbial alpha diversity, but there was an increased abundance of *Lactobacillaceae* and reduced abundance of *Clostridiaceae* in the cecum along with altered expression for three host genes in the ileum (Herrero-Encinas et al., 2020). Beyond considerations of dietary differences and broad measures of diversity (e.g., Aitchison distances), future studies may benefit from further work toward identifying and evaluating specific taxa based on previous reports and through the use of more enhanced methods for diversity analysis (e.g., sPLS-DA). In addition to employing more refined methods for diversity analysis, there remains a need for targeted prospective studies focusing on specific dietary modulations. These studies could help better characterize the probabilities of occurrence for the initially identified taxa.

A particular finding of our study is that treatment time (age) is associated with a stronger and more significant microbiota shift than differences in dietary treatment alone, and dietary treatment combined with treatment time is a significant factor as well. The control, olive, coconut, flaxseed, and fish oil diets yielded more distinctive taxonomic and functional differences that varied based on treatment time and age of the broiler.

### 4.2 PUFA-rich fat sources may promote host-beneficial cecal microbial taxa

The butyrate-producing *Dysosmobacter welbionis* species was found to be enriched in the fish oil group day 41 chickens. *Dysosmobacter welbionis* is generally identified as a beneficial type of gut bacteria known to positively associate with host metabolic health and alleviate inflammation (Roy, 2021). Other species enriched in the fish oil group were the SCFA-producing *Alistipes_A_871404 ihumii*, and butyrate-producing *Copromorpha sp900066305* and *Lachnoclostridium_A_130679 sp002160755* (*p* < 0.05). In addition, the lactate-fermenting, anaerobic taxa *Anaerotignum lactatifermentans*, and a potential biomarker for gut homeostasis, *Sellimonas intestinalis*, also showed association with the fish oil group at day 41 (*p* < 0.10). These results are similar to the reports from mammalian studies that have observed an increased abundance of SCFA-producing species as a result of PUFA-rich diets (Noriega et al., 2016; Watson et al., 2017), and PUFA-deficient diets that reduced SCFA production (Robertson et al., 2017). An additional effect of PUFA-rich diets on health in enriching SCFA-producing species is due to their ability to promote lactate producers, which then can cross-feed SCFA producers.

For the day 55 birds, predicted functions showed more alterations in the flaxseed oil and control groups. Enzymes predicted to be abundant in the flaxseed group include propionate kinase, which participates in the fermentation pathway that produces propionate. Other enzymes such as L-lactate dehydrogenase (cytochrome) and D-lactate dehydratase that participate in lactate fermentation and metabolism were also abundant in flaxseed, while microbial enzymes such as thiamine kinase and vitamin B12-transporting ATPase play important roles in transport and activation vitamins in bacterial cells, indicating their ability to produce vitamins B1 and B12, which is health promoting for the host. The flaxseed group is also found to have aminobutyraldehyde dehydrogenase in abundance. Aminobutyraldehyde dehydrogenase catalyzes the oxidation of 4-aminobutyraldehyde to 4-aminobutyric acid (GABA), which is a key step in the biosynthetic pathway of GABA neurotransmitter (Sarasa et al., 2020). This result indicates that gut microbes may have the capability to produce neurotransmitters that could regulate the gut–brain axis, thereby influencing host health.

As broilers have a shorter life span and are harvested before adulthood, this makes it challenging to identify clear dietary intervention effects separate from other confounders such as age while the microbiota is still developing. However, in our study, PUFA-rich dietary fat sources have shown signs of favorable microbial compositional and functional changes in broiler birds. The strategic inclusion of PUFA-rich fats in poultry feed could promote host-beneficial gut microbes, and this effect might be enhanced as the microbiota stabilizes with age. By aligning dietary PUFA fat sources with the natural microbial development timeline, there may be some benefit to broiler health and performance. Additional information on broiler performance is reported in Omaliko et al. (2024).

Although the inclusion of these dietary fats has promising potential for modulating gut microbiota and the use of 2–3% fish oil in the feed might elevate profit (Avi et al., 2023), it may also increase overall production costs (Zduńczyk and Jankowski, 2013). However, utilizing alternative sources, such as chia seed oil or camelina oil, or using whole seeds instead of extracted oils, may help reduce costs while still maintaining their beneficial effects on poultry health (Da Silva et al., 2024; Orczewska-Dudek and Pietras, 2019). Moreover, due to potential market trends relating to increased interest and demand for egg and meat products having higher levels of omega-3 fatty acids, the usage of dietary fat additives such as fish and flaxseed oil for chicken diets may be of substantial interest (Moghadam and Cherian, 2017). These trends include a projected market size increase for fortified eggs to amount to almost $500 million in the United States from 2023 to 2033 (Future Market Insights, 2023; Grand View Research, 2023). Additional investigation is needed to further verify the effect of PUFA-rich fat sources on broiler gut health and microbiota.

## 5 Conclusion

Our study suggests that the age of the chicken has a substantial impact on cecal microbial composition. In the case of dietary fats, PUFA-rich fat sources fish oil and flaxseed oils exhibited SCFA-producing taxa enrichment and microbial functions having an overall potential to contribute to broiler health.

## Data Availability

The data presented in the study are deposited in the NCBI SRA database Bioproject PRJNA1133683.

## References

[ref1] Abulizi N. Quin C. Brown K. Chan Y. K. Gill S. K. Gibson D. L. (2019). Gut mucosal proteins and bacteriome are shaped by the saturation index of dietary lipids. Nutrients 11:418. doi: 10.3390/nu11020418, PMID: 30781503 PMC6412740

[ref2] Al-Hilali A. (2018). Effect of dietary flaxseed oil on growth performance and serum lipid profiles in broilers. Pak. J. Nutr. 17, 512–517. doi: 10.3923/pjn.2018.512.517

[ref3] Alzueta C. Rodriguez M. L. Cutuli M. T. Rebole A. Ortiz L. T. Centeno C.. (2003). Effect of whole and demucilaged linseed in broiler chicken diets on digesta viscosity, nutrient utilisation and intestinal microflora. Br. Poult. Sci. 44, 67–74. doi: 10.1080/0007166031000085337, PMID: 12737228

[ref9001] Aviagen. (2022). Ross 708: Broiler management and nutrition specifications. Available at: https://aviagen.com/assets/Tech_Center/Ross_Broiler/Ross-roilerNutritionSpecifications2022-EN.pdf (Accessed June 5, 2023).

[ref4] Avi A. C. Islam M. T. Bari M. S. Khandoker R. S. S. Das G. B. (2023). Effects of fish oil on growth performance, carcass characteristics, blood parameter, and cost efficiency of broiler chicken. Adv. Anim. Vet. Sci. 11, 1083–1089. doi: 10.17582/journal.aavs/2023/11.7.1083.1089

[ref5] Aziza A. E. Awadin W. F. Quezada N. Cherian G. (2014). Gastrointestinal morphology, fatty acid profile, and production performance of broiler chickens fed camelina meal or fish oil. Eur. J. Lipid Sci. Technol. 116, 1727–1733. doi: 10.1002/ejlt.201400019

[ref6] Baião N. Lara L. (2005). Oil and fat in broiler nutrition. Braz. J. Poultry Sci. 7, 129–141. doi: 10.1590/S1516-635X2005000300001

[ref7] Barszcz M. Tuśnio A. Taciak M. (2024). Poultry nutrition. Phys. Sci. Rev. 9, 611–650. doi: 10.1515/psr-2021-0122

[ref8] Bokulich N. A. Kaehler B. D. Rideout J. R. Dillon M. Bolyen E. Knight R.. (2018). Optimizing taxonomic classification of marker-gene amplicon sequences with QIIME 2’s q2-feature-classifier plugin. Microbiome 6:90. doi: 10.1186/S40168-018-0470-Z, PMID: 29773078 PMC5956843

[ref9] Callahan B. J. McMurdie P. J. Rosen M. J. Han A. W. Johnson A. J. A. Holmes S. P. (2016). DADA2: high-resolution sample inference from Illumina amplicon data. Nat. Methods 13, 581–583. doi: 10.1038/nmeth.3869, PMID: 27214047 PMC4927377

[ref10] Cao L. Lê Cao K.-A. Boitard S. Besse P. (2011). Sparse PLS discriminant analysis: biologically relevant feature selection and graphical displays for multiclass problems. BMC Bioinf. 12, 1–17. doi: 10.1186/1471-2105-12-253 PMC3133555 21693065

[ref12] Da Silva A. Cabrera M. C. Olivero R. del Puerto M. Terevinto A. Saadoun A. (2024). The incorporation of chia seeds (*Salvia hispanica* L.) in the chicken diet promotes the enrichment of meat with n-3 fatty acids, particularly EPA and DHA. Appl. Food Res. 4:100416. doi: 10.1016/j.afres.2024.100416

[ref13] Emami N. K. Calik A. White M. B. Kimminau E. A. Dalloul R. A. (2020). Effect of probiotics and multi-component feed additives on microbiota, gut barrier and immune responses in broiler chickens during subclinical necrotic enteritis. Front. Vet. Sci. 7:572142. doi: 10.3389/fvets.2020.572142, PMID: 33324697 PMC7725796

[ref14] Estaki M. Jiang L. Bokulich N. A. McDonald D. González A. Kosciolek T.. (2020). QIIME 2 enables comprehensive end-to-end analysis of diverse microbiome data and comparative studies with publicly available data. Curr. Protoc. Bioinformatics 70:e100. doi: 10.1002/cpbi.100, PMID: 32343490 PMC9285460

[ref15] Fu Y. Wang Y. Gao H. Li D. Jiang R. Ge L.. (2021). Associations among dietary omega-3 polyunsaturated fatty acids, the gut microbiota, and intestinal immunity. Mediat. Inflamm. 2021, 1–11. doi: 10.1155/2021/8879227 PMC7801035 33488295

[ref16] Future Market Insights. (2023). Fortified eggs market size, share & trends to 2033. Available at: https://www.futuremarketinsights.com/reports/fortified-eggs-market (Accessed October 19, 2024).

[ref17] Grand View Research. (2023). Omega-3 market size, share & growth analysis report, 2030. Available at: https://www.grandviewresearch.com/industry-analysis/omega-3-market (Accessed October 19, 2024).

[ref18] Herrero-Encinas J. Blanch M. Pastor J. J. Mereu A. Ipharraguerre I. R. Menoyo D. (2020). Effects of a bioactive olive pomace extract from *Olea europaea* on growth performance, gut function, and intestinal microbiota in broiler chickens. Poult. Sci. 99, 2–10. doi: 10.3382/ps/pez467, PMID: 32416802 PMC7587805

[ref19] Józefiak D. Kierończyk B. Rawski M. Hejdysz M. Rutkowski A. Engberg R. M.. (2014). *Clostridium perfringens* challenge and dietary fat type affect broiler chicken performance and fermentation in the gastrointestinal tract. Animal 8, 912–922. doi: 10.1017/S1751731114000536, PMID: 24674938

[ref20] Kamran J. Mehmood S. Mahmud A. (2020). Effect of fat sources and emulsifier levels in broiler diets on performance, nutrient digestibility, and carcass parameters. Brazilian. J. Poultr. Sci. 22:eRBCA-2019. doi: 10.1590/1806-9061-2019-1158

[ref21] Knarreborg A. Simon M. A. Engberg R. M. Jensen B. B. Tannock G. W. (2002). Effects of dietary fat source and subtherapeutic levels of antibiotic on the bacterial community in the ileum of broiler chickens at various ages. Am Soc Microbiol 68, 5918–5924. doi: 10.1128/AEM.68.12.5918-5924.2002, PMID: 12450811 PMC134372

[ref22] Li X. Wu S. Li X. Yan T. Duan Y. Yang X.. (2018). Simultaneous supplementation of Bacillus subtilis and antibiotic growth promoters by stages improved intestinal function of pullets by altering gut microbiota. Front. Microbiol. 9:2328. doi: 10.3389/fmicb.2018.02328, PMID: 30369910 PMC6194165

[ref23] Lu M. Li R. W. Zhao H. Yan X. Lillehoj H. S. Sun Z.. (2020). Effects of Eimeria maxima and *Clostridium perfringens* infections on cecal microbial composition and the possible correlation with body weight gain in broiler chickens. Res. Vet. Sci. 132, 142–149. doi: 10.1016/j.rvsc.2020.05.013, PMID: 32575030

[ref24] Moghadam M. B. Cherian G. (2017). Use of flaxseed in poultry feeds to meet the human need for n-3 fatty acids. Worlds Poult. Sci. J. 73, 803–812. doi: 10.1017/S0043933917000721

[ref25] Neijat M. Habtewold J. Li S. Jing M. House J. D. (2020). Effect of dietary n-3 polyunsaturated fatty acids on the composition of cecal microbiome of Lohmann hens. Prostaglandins Leukot. Essent. Fat. Acids 162:102182. doi: 10.1016/j.plefa.2020.102182, PMID: 33038831

[ref26] Noriega B. S. Sanchez-Gonzalez M. A. Salyakina D. Coffman J. (2016). Understanding the impact of omega-3 rich diet on the gut microbiota. Case Rep. Med. 2016:9303. doi: 10.1155/2016/3089303, PMID: 27065349 PMC4808672

[ref27] Nova E. Gómez-Martinez S. González-Soltero R. (2022). The influence of dietary factors on the gut microbiota. Microorganisms 10:1368. doi: 10.3390/microorganisms10071368, PMID: 35889087 PMC9318379

[ref28] Omaliko P. C. Ferket P. R. Ogundare T. E. Apalowo O. O. Enenya I. G. Iwuozo O. C.. (2024). Impact of dietary fat types on expression levels of dopamine and serotonin transporters in the ileum of broiler chickens. Poult. Sci. 103:104114. doi: 10.1016/j.psj.2024.104114, PMID: 39214056 PMC11402036

[ref29] Orczewska-Dudek S. Pietras M. (2019). The effect of dietary *Camelina sativa* oil or cake in the diets of broiler chickens on growth performance, fatty acid profile, and sensory quality of meat. Animals 9:734. doi: 10.3390/ani9100734, PMID: 31569656 PMC6826988

[ref30] Plata G. Baxter N. T. Susanti D. Volland-Munson A. Gangaiah D. Nagireddy A.. (2022). Growth promotion and antibiotic induced metabolic shifts in the chicken gut microbiome. Commun. Biol. 5:293. doi: 10.1038/s42003-022-03239-6, PMID: 35365748 PMC8975857

[ref31] Precedence Research. (2023). Poultry feed market. Available at: https://www.precedenceresearch.com/poultry-feed-market (Accessed June 10, 2024).

[ref32] Robertson R. C. Oriach C. S. Murphy K. Moloney G. M. Cryan J. F. Dinan T. G.. (2017). Deficiency of essential dietary n-3 PUFA disrupts the caecal microbiome and metabolome in mice. Br. J. Nutr. 118, 959–970. doi: 10.1017/S0007114517002999, PMID: 29173237

[ref33] Roy L. (2021). Gut microbiota Dysosmobacter welbionis is a newly isolated human commensal bacterium preventing diet-induced obesity and metabolic disorders in mice. Gut 71, 534–543. doi: 10.1136/gutjnl-2020-323778, PMID: 34108237 PMC8862106

[ref34] Rychlik I. (2020). Composition and function of chicken gut microbiota. Animals 10:103. doi: 10.3390/ani10010103, PMID: 31936291 PMC7022619

[ref35] Salsinha A. S. Cima A. Araújo-Rodrigues H. Viana S. Reis F. Coscueta E. R.. (2024). The use of an in vitro fecal fermentation model to uncover the beneficial role of omega-3 and punicic acid in gut microbiota alterations induced by a Western diet. Food Funct. 15, 6095–6117. doi: 10.1039/D4FO00727A, PMID: 38757812

[ref36] Sarasa S. B. Mahendran R. Muthusamy G. Thankappan B. Selta D. R. F. Angayarkanni J. (2020). A brief review on the non-protein amino acid, gamma-amino butyric acid (GABA): its production and role in microbes. Curr. Microbiol. 77, 534–544. doi: 10.1007/s00284-019-01839-w, PMID: 31844936

[ref37] Schoeler M. Caesar R. (2019). Dietary lipids, gut microbiota and lipid metabolism. Rev. Endocr. Metab. Disorders 20, 461–472. doi: 10.1007/s11154-019-09512-0, PMID: 31707624 PMC6938793

[ref38] Takeshita N. Watanabe T. Ishida-Kuroki K. Sekizaki T. (2021). Transition of microbiota in chicken cecal droppings from commercial broiler farms. BMC Vet. Res. 17, 1–11. doi: 10.1186/s12917-020-02688-7 33407476 PMC7789685

[ref39] Watson H. Mitra S. Croden F. C. Taylor M. Wood H. M. Perry S. L.. (2017). A randomised trial of the effect of omega-3 polyunsaturated fatty acid supplements on the human intestinal microbiota. Gut 67, 1974–1983. doi: 10.1136/gutjnl-2017-314968, PMID: 28951525

[ref40] Williams T. J. Athrey G. N. (2020). Cloacal swabs are unreliable sources for estimating lower gastro-intestinal tract microbiota in chicken. Microorganisms 8:718. doi: 10.21203/rs.2.23791/v1 32408567 PMC7285018

[ref41] Wolters M. Ahrens J. Romaní-Pérez M. Watkins C. Sanz Y. Benítez-Páez A.. (2019). Dietary fat, the gut microbiota, and metabolic health – a systematic review conducted within the MyNewGut project. Clin. Nutr. 38, 2504–2520. doi: 10.1016/j.clnu.2018.12.024 30655101

[ref42] Yang Q. Wang S. Ji Y. Chen H. Zhang H. Chen W.. (2017). Dietary intake of n-3 PUFAs modifies the absorption, distribution and bioavailability of fatty acids in the mouse gastrointestinal tract. Lipids Health Dis. 16, 10–18. doi: 10.1186/s12944-016-0399-9, PMID: 28095863 PMC5240384

[ref43] Yin Z. Ji S. Yang J. Guo W. Li Y. Ren Z.. (2023). Cecal microbial succession and its apparent association with nutrient metabolism in broiler chickens. Msphere 8, e00614–e00622. doi: 10.1128/msphere.00614-22 37017520 PMC10286727

[ref44] Zduńczyk Z. Jankowski J. (2013). Poultry meat as functional food: modification of the fatty acid profile-a review. Annal. Anim. Sci. 13, 463–480. doi: 10.2478/aoas-2013-0039

[ref45] Zeitz J. O. Fennhoff J. Kluge H. Stangl G. I. Eder K. (2015). Effects of dietary fats rich in lauric and myristic acid on performance, intestinal morphology, gut microbes, and meat quality in broilers. Poult. Sci. 94, 2404–2413. doi: 10.3382/PS/PEV191, PMID: 26240391

[ref46] Zhu Q. Sun P. Zhang B. Kong L. Xiao C. Song Z. (2021). Progress on gut health maintenance and antibiotic alternatives in broiler chicken production. Front. Nutr. 8:692839. doi: 10.3389/fnut.2021.692839, PMID: 34869510 PMC8636040

