# Supplementary material for: Effect of dietary fat source on the composition of the cecal microbiome in maturing broiler chicken
Source: Front Microbiol. 2024 Nov 27;15:1462757. doi: 10.3389/fmicb.2024.1462757 (PMC11631920; doi:10.3389/fmicb.2024.1462757)
Supplement: Supplementary file 2 [file Data_Sheet_1.pdf]

Supplementary Figure 1.

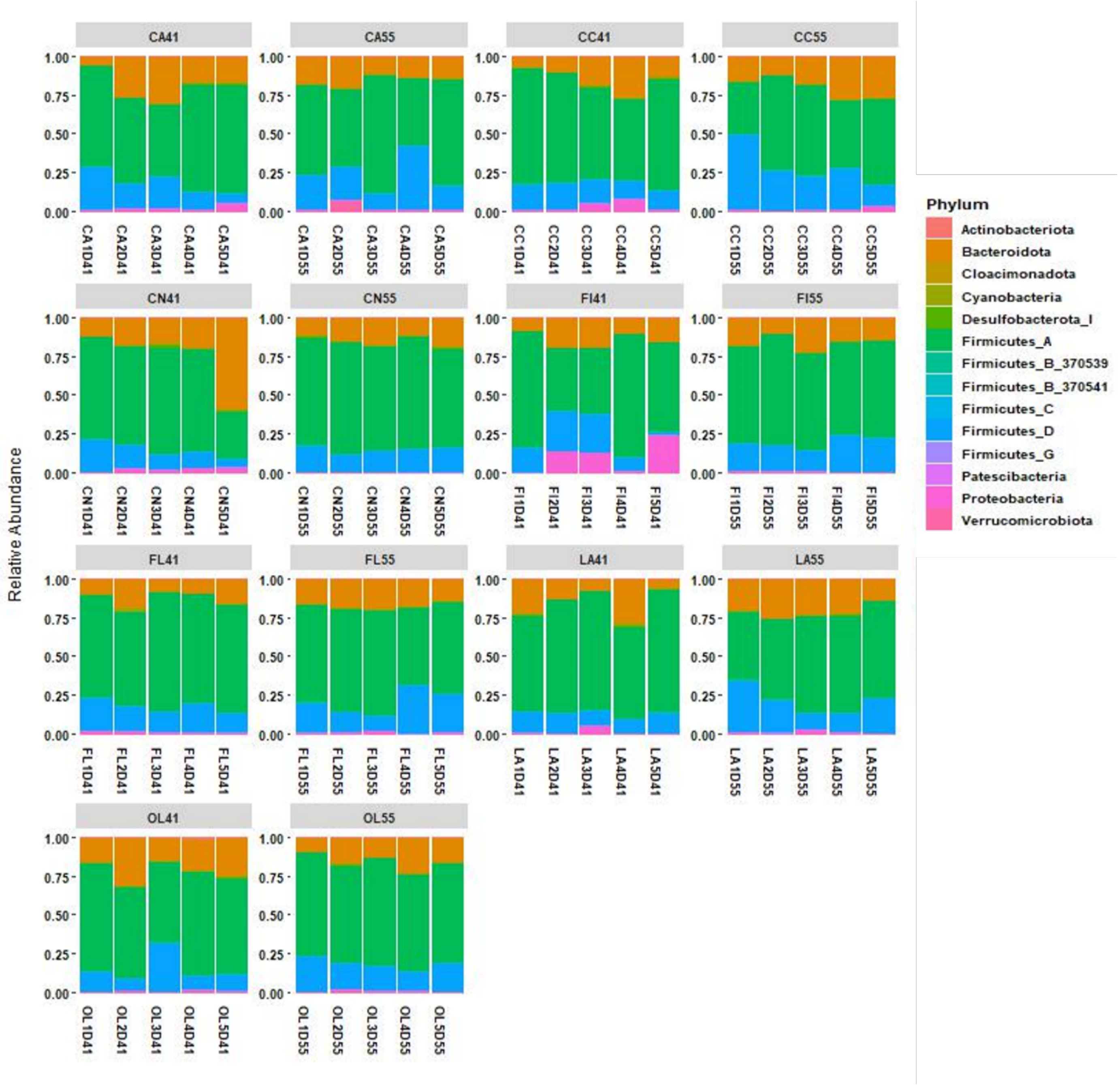

Stack bar plot showing phylum level relative abundance plot comparing day 41 treatment group profile with day 55.
CA: Canola oil; CC: Coconut oil; CN: Control; FI: Fish oil; FL: Flaxseed oil; LA: Lard; OL: Olive oil

Supplementary Figure 2A.

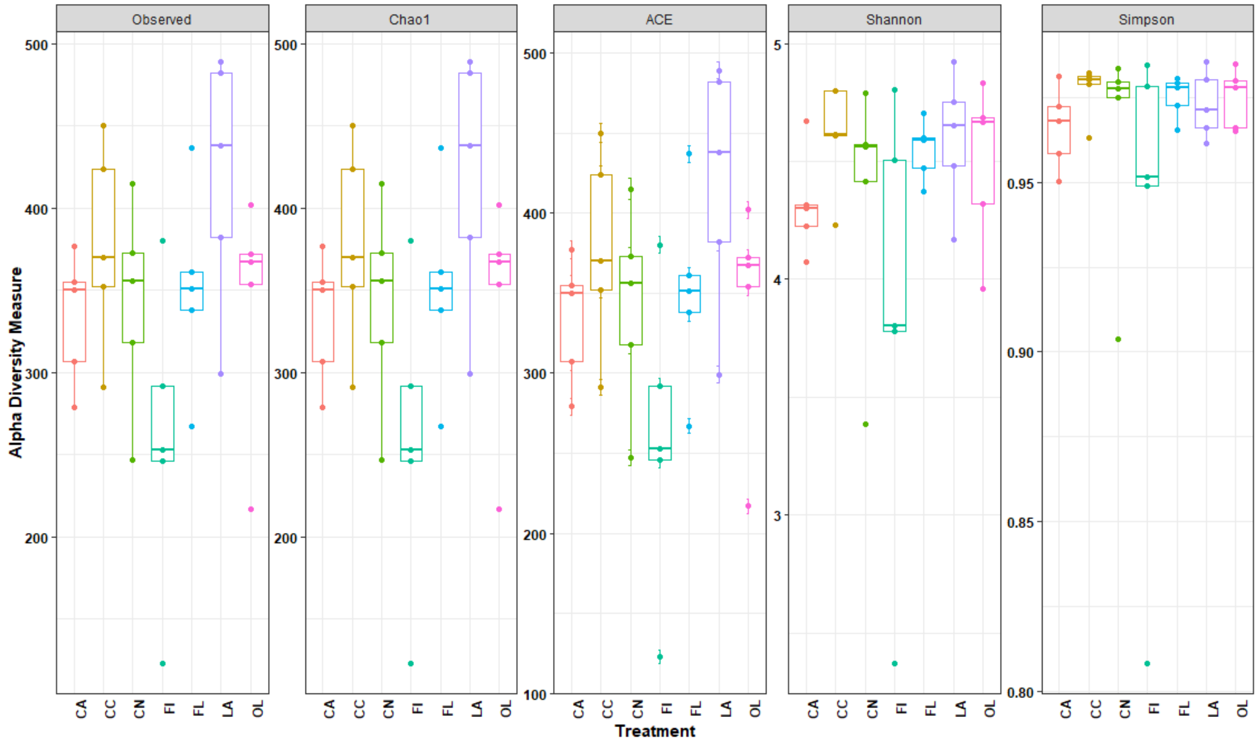

Supplementary Figure 2B.

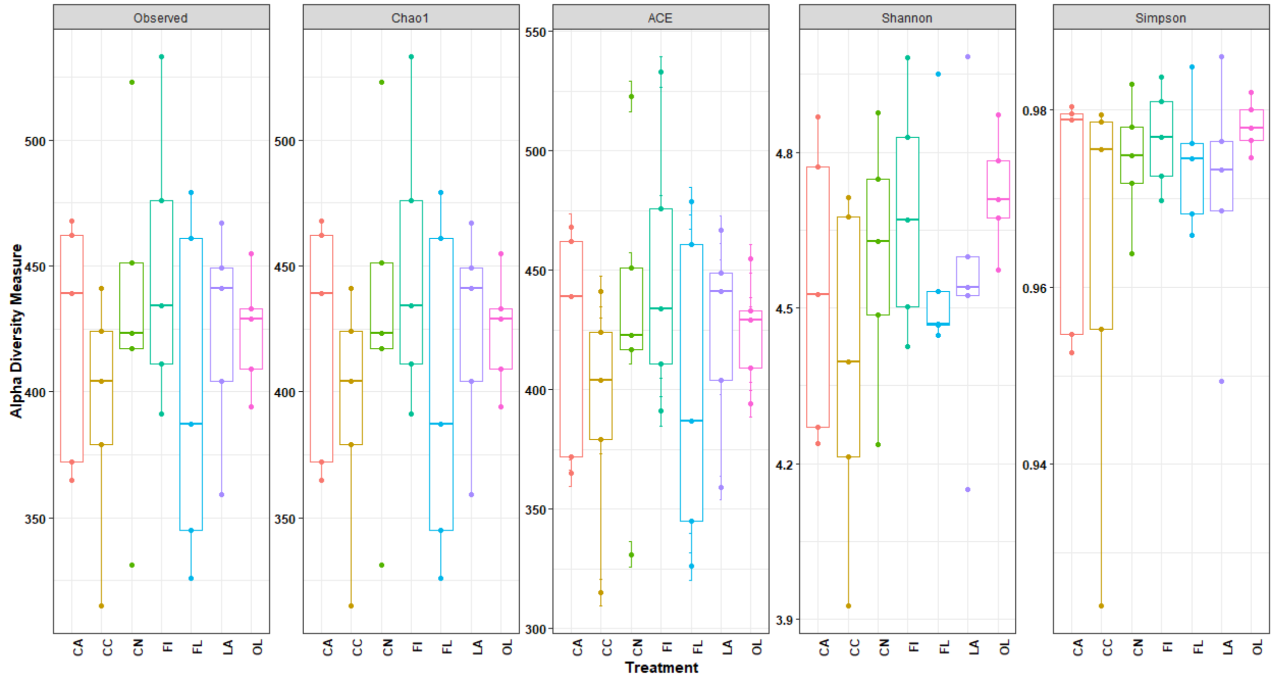

ASV-level alpha diversity indices across treatment groups. (A) Observed, Chao1, ACE, Shannon and Simpson indices for day 41 samples. (B) Observed, Chao1, ACE, Shannon and Simpson indices for day 55 samples.
CA: Canola oil; CC: Coconut oil; CN: Control; FI: Fish oil; FL: Flaxseed oil; LA: Lard; OL: Olive oil

Supplementary Figure 3A.

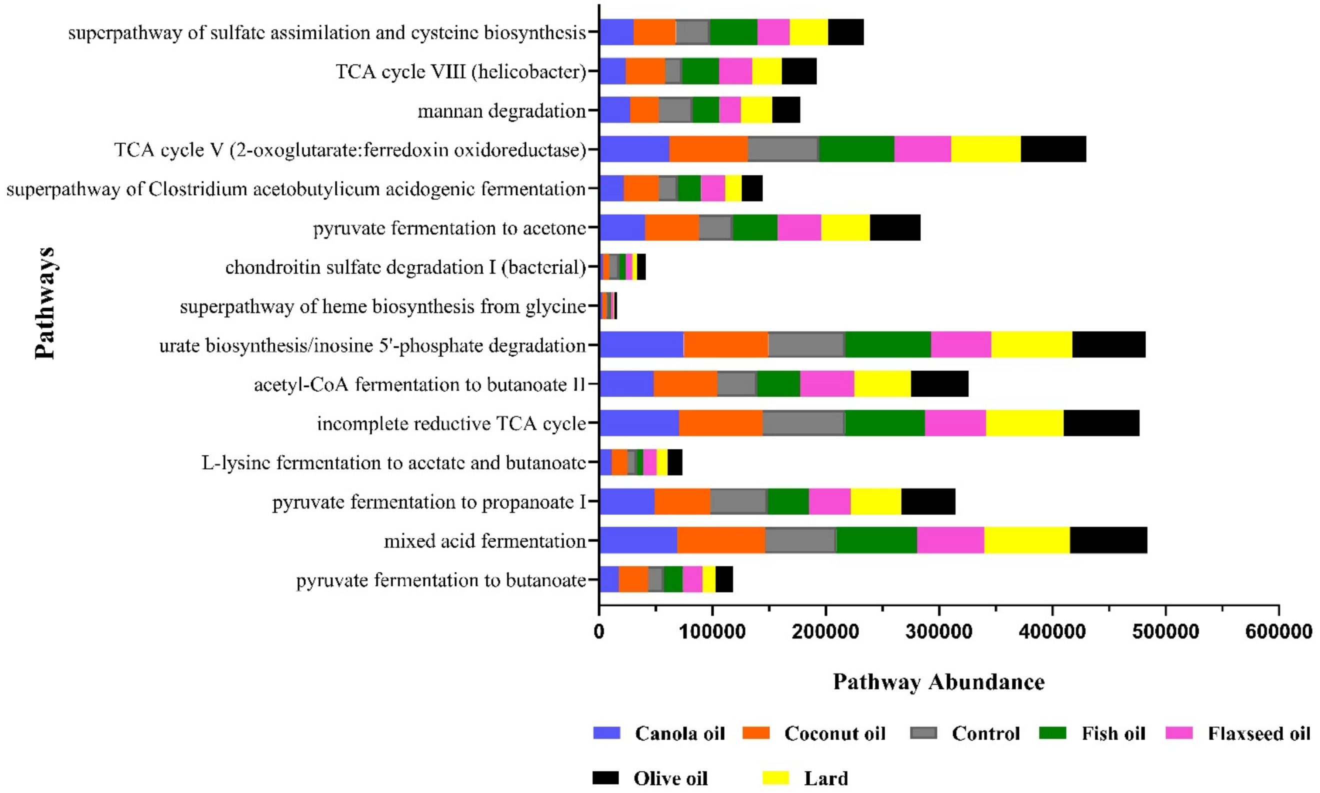

Supplementary Figure 3B.

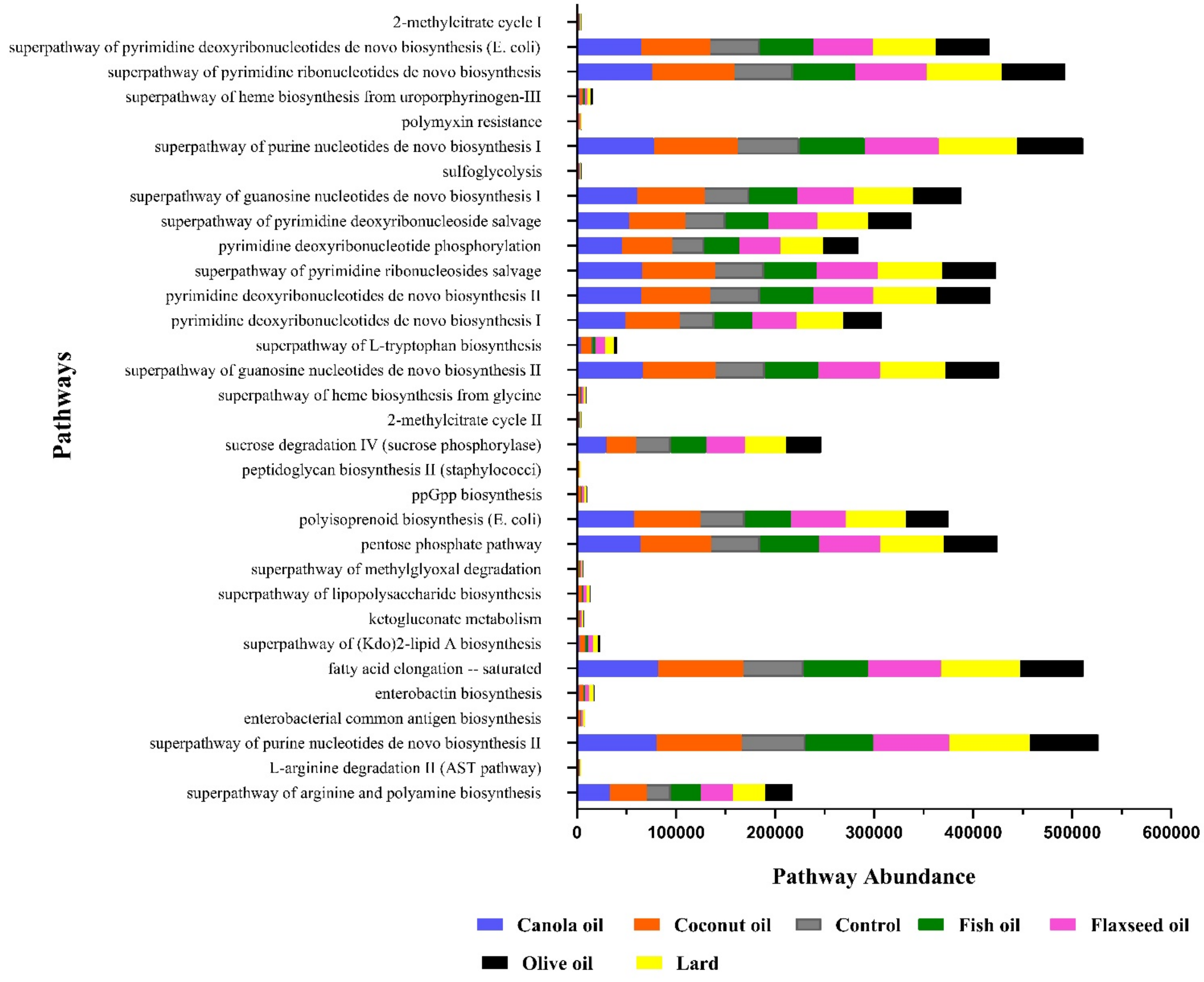

Stack bar plot indicating absolute abundances for ALDEx2 identified pathways. (A) Day 41 differentially abundant pathways; (B) Day 55 differentially abundant pathways.
