# Supplementary material for: Effect of dietary fat source on the composition of the cecal microbiome in maturing broiler chicken
Source: Front Microbiol. 2024 Nov 27;15:1462757. doi: 10.3389/fmicb.2024.1462757 (PMC11631920; doi:10.3389/fmicb.2024.1462757)
Supplement: Supplementary file 3 [file Data_Sheet_2.PDF]

Table 1. Fatty acid composition of fat types used in diets (% of total fatty acids).[1]

Fatty acid	Canola oil	Coconut oil	Fish oil	Flaxseed oil	Lard	Olive oil
C8:0 Caprylic	<0.02	9.54	<0.02	<0.02	<0.02	<0.02
C10:0 Capric	<0.02	5.94	<0.02	<0.02	0.08	<0.02
C12:0 Lauric	<0.02	44.17	0.10	<0.02	0.08	<0.02
C14:0 Myristic	0.05	16.14	5.80	0.05	1.34	<0.02
C16:0 Palmitic	3.61	6.25	13.96	4.84	24.14	10.66
C16:1 Palmitoleic	0.28	<0.04	9.18	0.10	1.83	0.95
C18:0 Stearic	1.41	2.49	2.93	3.25	16.51	3.50
C18:1 Oleic	57.85	4.34	9.73	17.04	33.31	70.68
C18:2 Linoleic	17.67	0.73	2.10	14.04	12.56	5.81
C18:3 α-linolenic	6.30	<0.02	0.85	50.14	0.67	0.66
C20:4 Arachidonic	<0.02	0.03	0.91	<0.02	0.23	<0.02
C20:5 Eicosapentaenoic	<0.02	<0.02	14.27	<0.02	<0.02	<0.02
C22:6 Docosahexaenoic	<0.02	<0.02	9.92	<0.02	0.03	<0.02
Fatty acid characteristics						
Saturated	6.20	85.58	24.45	8.53	42.89	14.81
Monounsaturated	61.26	4.38	21.28	17.36	35.70	71.93
Polyunsaturated	24.17	0.78	37.52	64.33	14.23	6.59

[1]Fat types were analyzed for fatty acid composition by Eurofins Scientific Inc. Nutrient Analysis Center, 2200 Rittenhouse Street, Suite 150, Des Moines, IA 50321.

Table 2. Composition of Experimental Starter Diets (% "as is") [1].

Ingredients	CN	FL	CA	FI	CC	OL	LA
Corn	53.22	53.22	53.22	53.22	53.22	53.22	53.22
Soybean Meal	39.40	39.40	39.40	39.40	39.40	39.40	39.40
Fat/Oil*	3.00	3.00	3.00	3.00	3.00	3.00	3.00
Mono-Dicalcium Phosphate	1.81	1.81	1.81	1.81	1.81	1.81	1.81
Limestone 37%	0.95	0.95	0.95	0.95	0.95	0.95	0.95
Salt NaCl	0.45	0.45	0.45	0.45	0.45	0.45	0.45
DL-Methionine	0.35	0.35	0.35	0.35	0.35	0.35	0.35
NCSU Poultry Mineral Premix[2]	0.20	0.20	0.20	0.20	0.20	0.20	0.20
Choline Chloride 60%	0.20	0.20	0.20	0.20	0.20	0.20	0.20
L-Lysine	0.18	0.18	0.18	0.18	0.18	0.18	0.18
L-Threonine	0.09	0.09	0.09	0.09	0.09	0.09	0.09
NCSU Poultry Vitamin Premix[3]	0.05	0.05	0.05	0.05	0.05	0.05	0.05
Selenium Premix[+]	0.05	0.05	0.05	0.05	0.05	0.05	0.05
Santoquin	0.05	0.05	0.05	0.05	0.05	0.05	0.05
Analyzed nutrient composition							
Metabolizable Energy (Kcal/kg)	1,433	1,429	1,438	1,423	1,432	1,412	1,411
Crude Protein, %	23.06	23.19	21.88	22.63	22.75	24.38	23.25
Crude Fat, %	5.42	5.12	5.46	5.27	5.25	5.18	5.05
Crude Fiber, %	2.1	2.3	2.3	2.3	2.5	2.3	2.3
Ash, %	5.64	5.55	5.66	5.57	5.30	5.73	5.77
Calculated nutrient composition							
Total Sulfur Amino Acids, %	0.19	0.19	0.19	0.19	0.19	0.19	0.19
Lysine, %	1.44	1.44	1.44	1.44	1.44	1.44	1.44
Calcium, %	0.96	0.96	0.96	0.96	0.96	0.96	0.96
Available phosphorus, %	0.48	0.48	0.48	0.48	0.48	0.48	0.48

[1]Diets used in the study included the following: i) conventional Corn-soybean meal (SBM) with the addition of poultry fat as fat type (CN diet); ii) conventional corn-SBM with Flax Seed oil as fat type (FL); iii) conventional Corn-SBM with the addition of Canola oil as the fat type (CA); iv) conventional Corn-SBM with Fish oil was the fat type (FI); v) consist of conventional Corn-SBM with addition of Coconut oil as the fat type (CC); vi) conventional Corn-SBM in which Olive oil was incorporated as fat type (OL); vii) conventional Corn-SBM with Lard used as the fat type (LA). Each of these 7 diets were separately formulated for the starter (d 1 to 21) phase of experiment.

*7 different fat types were added at 3% in each diet.

[2]Mineral Premix, supplied per kilogram of diet: Manganese (Mn), 60 mg; Zinc (Zn), 60 mg; Iron (Fe), 40 mg; Copper (Cu), 5 mg; Iodine (I), 1.2mg; Cobalt (Co), 0.5 mg.

[3]Vitamin Premix, supplied per kilogram of diet: Vitamin A (6,600 IU), Vitamin D (1,980 IU), Vitamin E (33 IU), Vitamin B12 (0.02 mg), Biotin (0.13 mg), Menadione (1.98 mg), Thiamine (1.98 mg), Riboflavin (6.60 mg), d-Pantothenic Acid (11.0 mg), Vitamin B6 (3.96 mg), Niacin (55.0 mg), Folic Acid (1.1 mg).

[4]Experimental diets were analyzed for proximate nutrient composition by Eurofins Scientific Inc. Nutrient Analysis Center, 2200 Rittenhouse Street, Suite 150, Des Moines, IA 50321.

[+]Selenium Premix provides 0.3 mg Selenium/Kg of feed as sodium selenite.

Table 3. Composition of Experimental Grower Diets (% "as is"). [1]

Ingredients	CN	FL	CA	FI	CC	OL	LA
Corn	58.84	53.22	53.22	53.22	53.22	53.22	53.22
Soybean Meal	32.75	39.40	39.40	39.40	39.40	39.40	39.40
Fat/Oil*	3.00	3.00	3.00	3.00	3.00	3.00	3.00
Mono-Dicalcium Phosphate	1.81	1.81	1.81	1.81	1.81	1.81	1.81
Limestone 37%	0.95	0.95	0.95	0.95	0.95	0.95	0.95
Salt NaCl	0.45	0.45	0.45	0.45	0.45	0.45	0.45
DL-Methionine	0.35	0.35	0.35	0.35	0.35	0.35	0.35
NCSU Poultry Mineral Premix[2]	0.20	0.20	0.20	0.20	0.20	0.20	0.20
Choline Chloride 60%	0.20	0.20	0.20	0.20	0.20	0.20	0.20
L-Lysine	0.18	0.18	0.18	0.18	0.18	0.18	0.18
L-Threonine	0.09	0.09	0.09	0.09	0.09	0.09	0.09
NCSU Poultry Vitamin Premix[3]	0.05	0.05	0.05	0.05	0.05	0.05	0.05
Selenium Premix[+]	0.05	0.05	0.05	0.05	0.05	0.05	0.05
Santoquin	0.05	0.05	0.05	0.05	0.05	0.05	0.05
Analyzed nutrient composition							
Metabolizable Energy (Kcal/kg)	1,443	1,447	1,457	1,446	1,458	1,455	1,436
Crude Protein, %	23.31	22.81	21.81	23.63	22.13	22.81	22.25
Crude Fat, %	5.04	5.37	5.40	5.29	5.44	5.40	5.22
Crude Fiber, %	2.0	2.0	2.0	2.2	2.1	2.1	2.0
Ash, %	4.85	4.95	5.03	4.98	4.66	5.17	4.99
Calculated nutrient composition							
Total Sulfur Amino Acids, %	0.17	0.17	0.17	0.17	0.17	0.17	0.17
Lysine, %	1.30	1.30	1.30	1.30	1.30	1.30	1.30
Calcium, %	0.87	0.87	0.87	0.87	0.87	0.87	0.87
Available phosphorus, %	0.44	0.44	0.44	0.44	0.44	0.44	0.44

[1]Diets used in the study included the following: i) conventional Corn-soybean meal (SBM) with the addition of poultry fat as fat type (CN diet); ii) conventional corn-SBM with Flax Seed oil as fat type (FL); iii) conventional Corn-SBM with the addition of Canola oil as the fat type (CA); iv) conventional Corn-SBM with Fish oil was the fat type (FI); v) consist of conventional Corn-SBM with addition of Coconut oil as the fat type (CC); vi) conventional Corn-SBM in which Olive oil was incorporated as fat type (OL); vii) conventional Corn-SBM with Lard used as the fat type (LA). Each of these 7 diets were separately formulated for the starter (d 1 to 21) phase of experiment.

*7 different fat types were added at 3% in each diet.

[2]Mineral Premix, supplied per kilogram of diet: Manganese (Mn), 60 mg; Zinc (Zn), 60 mg; Iron (Fe), 40 mg; Copper (Cu), 5 mg; Iodine (I), 1.2mg; Cobalt (Co), 0.5 mg.

[3]Vitamin Premix, supplied per kilogram of diet: Vitamin A (6,600 IU), Vitamin D (1,980 IU), Vitamin E (33 IU), Vitamin B12 (0.02 mg), Biotin (0.13 mg), Menadione (1.98 mg), Thiamine (1.98 mg), Riboflavin (6.60 mg), d-Pantothenic Acid (11.0 mg), Vitamin B6 (3.96 mg), Niacin (55.0 mg), Folic Acid (1.1 mg).

[4]Experimental diets were analyzed for proximate nutrient composition by Eurofins Scientific Inc. Nutrient Analysis Center, 2200 Rittenhouse Street, Suite 150, Des Moines, IA 50321.

[+]Selenium Premix provides 0.3 mg Selenium/Kg of feed as sodium selenite.

Table 4. Composition of Experimental Finisher Diets (% “as is”). [1]

Ingredients	CN	FL	CA	FI	CC	OL	LA
Corn	53.22	53.22	53.22	53.22	53.22	53.22	53.22
Soybean Meal	39.40	39.40	39.40	39.40	39.40	39.40	39.40
Fat/Oil*	3.00	3.00	3.00	3.00	3.00	3.00	3.00
Mono-Dicalcium Phosphate	1.81	1.81	1.81	1.81	1.81	1.81	1.81
Limestone 37%	0.95	0.95	0.95	0.95	0.95	0.95	0.95
Salt NaCl	0.45	0.45	0.45	0.45	0.45	0.45	0.45
DL-Methionine	0.35	0.35	0.35	0.35	0.35	0.35	0.35
NCSU Poultry Mineral Premix[2]	0.20	0.20	0.20	0.20	0.20	0.20	0.20
Choline Chloride 60%	0.20	0.20	0.20	0.20	0.20	0.20	0.20
L-Lysine	0.18	0.18	0.18	0.18	0.18	0.18	0.18
L-Threonine	0.09	0.09	0.09	0.09	0.09	0.09	0.09
NCSU Poultry Vitamin Premix[3]	0.05	0.05	0.05	0.05	0.05	0.05	0.05
Selenium Premix[+]	0.05	0.05	0.05	0.05	0.05	0.05	0.05
Santoquin	0.05	0.05	0.05	0.05	0.05	0.05	0.05
Analyzed nutrient composition							
Metabolizable Energy (Kcal/kg)	1,449	1,447	1,450	1,467	1,445	1,430	1,487
Crude Protein, %	21.31	21.13	21.38	21.44	20.13	21.13	18.81
Crude Fat, %	5.27	5.49	5.32	5.21	5.30	5.29	5.68
Crude Fiber, %	2.1	2.0	2.1	1.9	2.0	2.0	1.9
Ash, %	4.60	4.64	4.39	4.49	4.21	4.48	3.97
Calculated nutrient composition							
Total Sulfur Amino Acids, %	0.15	0.15	0.15	0.15	0.15	0.15	0.15
Lysine, %	1.21	1.21	1.21	1.21	1.21	1.21	1.21
Calcium, %	0.81	0.81	0.81	0.81	0.81	0.81	0.81
Available phosphorus, %	0.40	0.40	0.40	0.40	0.40	0.40	0.40

[1]Diets used in the study included the following: i) conventional Corn-soybean meal (SBM) with the addition of poultry fat as fat type (CN diet); ii) conventional corn-SBM with Flax Seed oil as fat type (FL); iii) conventional Corn-SBM with the addition of Canola oil as the fat type (CA); iv) conventional Corn-SBM with Fish oil was the fat type (FI); v) consist of conventional Corn-SBM with addition of Coconut oil as the fat type (CC); vi) conventional Corn-SBM in which Olive oil was incorporated as fat type (OL); vii) conventional Corn-SBM with Lard used as the fat type (LA). Each of these 7 diets were separately formulated for the starter (d 1 to 21) phase of experiment.

*7 different fat types were added at 3% in each diet.

[2]Mineral Premix, supplied per kilogram of diet: Manganese (Mn), 60 mg; Zinc (Zn), 60 mg; Iron (Fe), 40 mg; Copper (Cu), 5 mg; Iodine (I), 1.2mg; Cobalt (Co), 0.5 mg.

[3]Vitamin Premix, supplied per kilogram of diet: Vitamin A (6,600 IU), Vitamin D (1,980 IU), Vitamin E (33 IU), Vitamin B12 (0.02 mg), Biotin (0.13 mg), Menadione (1.98 mg), Thiamine (1.98 mg), Riboflavin (6.60 mg), d-Pantothenic Acid (11.0 mg), Vitamin B6 (3.96 mg), Niacin (55.0 mg), Folic Acid (1.1 mg).

[4]Experimental diets were analyzed for proximate nutrient composition by Eurofins Scientific Inc. Nutrient Analysis Center, 2200 Rittenhouse Street, Suite 150, Des Moines, IA 50321.

[+]Selenium Premix provides 0.3 mg Selenium/Kg of feed as sodium selenite.

Table 5. Genus level alpha diversity (Shannon index)

Treatment	Day 41	Day 55	*P* -value
Canola oil	3.43 ± 0.12	3.51 ± 0.24	0.49
Coconut oil	3.63 ± 0.16	3.33 ± 0.38	0.14
Fish oil	3.09 ± 0.64	3.65 ± 0.17	0.09
Flaxseed oil	3.53 ± 0.15	3.59 ± 0.16	0.53
Lard	3.52 ± 0.27	3.56 ± 0.23	0.83
Olive oil	3.54 ± 0.21	3.70 ± 0.09	0.17
Day 41 Control vs Treatment			
	Control	Treatment	*P* -value
CN vs CA	3.41 ± 0.39	3.43 ± 0.12	0.95
CN vs CC	3.41 ± 0.39	3.63 ± 0.15	0.27
CN vs FI	3.41 ± 0.39	3.09 ± 0.64	0.35
CN vs FL	3.41 ± 0.39	3.53 ± 0.15	0.55
CN vs LA	3.41 ± 0.39	3.52 ± 0.27	0.62
CN vs OL	3.41 ± 0.39	3.54 ± 0.21	0.53

Day 55 Control vs Treatment			
	Control	Treatment	*P* -value
CN vs CA	3.63 ± 0.17	3.51 ± 0.24	0.41
CN vs CC	3.63 ± 0.17	3.33 ± 0.38	0.14
CN vs FI	3.63 ± 0.17	3.65 ± 0.17	0.88
CN vs FL	3.63 ± 0.17	3.59 ± 0.16	0.74
CN vs LA	3.63 ± 0.17	3.56 ± 0.23	0.60
CN vs OL	3.63 ± 0.17	3.70 ± 0.09	0.48

CA: Canola oil; CC: Coconut oil; CN: Control; FI: Fish oil; FL: Flaxseed oil; LA: Lard; OL: Olive oil
The data are expressed as means ±SD, with $n = 5$ per treatment.

Table 6. Differentially abundant taxa

Day 41				Day 55			
Taxa Name	KW *P*-value	Sig. Pair Dunn. Test	*P* adj (Bonferroni)	Taxa Name	KW *P*-value	Sig. Pair Dunn. Test	*P* adj (Bonferroni)
Family CAG-508 (class *Clostridia*)	0.02	↑ FL ↓ CA	0.002	*Ruminococcaceae*	0.011	↑ CN ↓ LA	0.012
Dysosmobacter welbionis	0.029	↑ FI ↓ CN	0.009			↑ CN ↓ OL	0.003
Faecousia sp000434635	0.035	↑ OL ↓ FI	0.017	*Coprobacillaceae,*	0.008	↑ OL ↓ CA	0.0081
Copromorpha sp900066305	0.040	↑ FI ↓ LA	0.026			↑ FL ↓ CA	0.011
Alistipes_A_871404 ihumii	0.051	↑ FI ↓ LA	0.014	*Mammaliicoccus lentus*	0.027	↑ LA ↓ OL	0.005
Lachnoclostridium_A_130679 sp.	0.051	↑ FI ↓ LA	0.039	*Blautia_A_141781*	0.039	↑ CC ↓ OL	0.026
Gemmiger_A_73276 avium	0.039	↑ OL ↓ FL	0.023	*Dehalobacteriaceae*	0.010	↑ CN ↓ FI	0.010
Anaerotignum lactatifermentans	0.046	↑ FI ↓ FL	0.047			↑ CN ↓ OL	0.032
				Mediterraneibacter_A_155507	0.032	↑ FL ↓ FI	0.009
				Holdemania	0.015	↑ FL ↓ CA	0.029
				UBA5905(Acutalibacteraceae)	0.007	↑ CN ↓ OL	0.017
						↑ CN ↓ FI	0.005

CA: Canola oil; CC: Coconut oil; CN: Control; FI: Fish oil; FL: Flaxseed oil; LA: Lard; OL: Olive oil
